# Comparative Genomics Identifies the Mouse *Bmp3* Promoter and an Upstream Evolutionary Conserved Region (ECR) in Mammals

**DOI:** 10.1371/journal.pone.0057840

**Published:** 2013-02-22

**Authors:** Jonathan W. Lowery, Anna W. LaVigne, Shoichiro Kokabu, Vicki Rosen

**Affiliations:** Department of Developmental Biology, Harvard School of Dental Medicine, Boston, Massachusetts, United States of America; University of South Florida, United States of America

## Abstract

The Bone Morphogenetic Protein (BMP) pathway is a multi-member signaling cascade whose basic components are found in all animals. One member, BMP3, which arose more recently in evolution and is found only in deuterostomes, serves a unique role as an antagonist to both the canonical BMP and Activin pathways. However, the mechanisms that control BMP3 expression, and the *cis*-regulatory regions mediating this regulation, remain poorly defined. With this in mind, we sought to identify the *Bmp3* promoter in mouse *(M. musculus*) through functional and comparative genomic analyses. We found that the minimal promoter required for expression in resides within 0.8 kb upstream of *Bmp3* in a region that is highly conserved with rat (*R. norvegicus*). We also found that an upstream region abutting the minimal promoter acts as a repressor of the minimal promoter in HEK293T cells and osteoblasts. Strikingly, a portion of this region is conserved among all available eutherian mammal genomes (47/47), but not in any non-eutherian animal (0/136). We also identified multiple conserved transcription factor binding sites in the *Bmp3* upstream ECR, suggesting that this region may preserve common *cis*-regulatory elements that govern *Bmp3* expression across eutherian mammals. Since dysregulation of BMP signaling appears to play a role in human health and disease, our findings may have application in the development of novel therapeutics aimed at modulating BMP signaling in humans.

## Introduction

The Bone Morphogenetic Protein (BMP) pathway is a signaling cascade that has ancient origins in the evolution of animals, arising 1.2–1.4 billion years ago [1], [2]. Canonical BMP signaling occurs through BMP ligand interaction with a complex of type I and type II BMP receptors, leading to activation of a class of downstream transcription factors (SMADs in vertebrates, MAD in *Drosophila*, SMA in *C. elegans*). Strikingly, this basic mechanism is highly conserved across all animals [2] and, as no non-animal counterparts have been identified, the BMP pathway is likely a key advancement in the evolution of animals.

Although the first observation of BMP activity in mammals was its ability to induce ectopic bone formation [3], BMP signaling has since been implicated in the development of nearly all vertebrate organs and is required for some of the earliest developmental processes, including gastrulation and axis determination [4], [5]. Thus, it is not surprising that BMP signaling is tightly regulated at many levels. For instance, extracellular antagonists that sequester BMP ligands away from BMP receptors (eg, Noggin) and E3-ubiquitin ligases (eg, SMURF1) that promote degradation of BMP receptors and SMADs [6], [7] are ancestral mechanisms for reducing BMP pathway activation that are conserved as early as sponges [2].

Arising more recently in evolution, the BMP ligand BMP3 serves a unique function by antagonizing the canonical BMP and Activin pathways. Homologs of BMP3 have only been identified in deuterostomes, but are present as early as echinoderms (sea urchin [8]) and hemichordates (acorn worm (Acorn Worm Genome Project, Baylor). Though the mature domains of the prototypical BMP ligands BMP2/4 and BMP5/6/7 (Dpp and Gbb in *Drosophila*, respectively) share dramatic identity, BMP3 is highly divergent from other BMP ligands in that it falls into an intermediate phylogenetic clade between TGF-β/Activin and BMP ligands [9], [10] and shares only 40% amino acid identity with the ancestral BMP2/4 and BMP5/6/7 groups [11]. For many years after its identification, mammalian BMP3 was thought to function like a typical BMP ligand [12], [13]. However, more recent *in vivo* analyses suggest that BMP3 serves an inhibitory function. For instance, while BMP ligands promote osteogenesis [14], *Bmp3* knockout mice have high bone mass, indicating that BMP3 acts as a negative regulator of osteogenesis *in vivo* [15]. Moreover, BMP3 inhibits BMP2-induced differentiation of osteoprogenitors into osteoblasts, the cells which produce bone matrix [15], [16], [17], [18]. These findings have been extended to overexpression studies in chick [19], *Xenopus* [20], [21], and mouse [22], all of which consistently indicate that BMP3 negatively regulates the BMP and Activin pathways. While the precise mechanism for this inhibition remains unclear, BMP3 has been demonstrated to both sequester BMP receptors into inactive signaling complexes [15], [20] through high affinity interaction with the receptor ACVR2B [17], [23], [24] and lead to altered TGF-β/Activin signaling [15], [16], [24], [25], [26], which commonly antagonizes BMP-mediated effects [27], [28], [29], [30], [31], [32], [33], [34], [35], [36], [37], [38].

Despite significant progress in distinguishing BMP3 as a unique inhibitory ligand among BMPs, the mechanisms that regulate BMP3 expression are unclear. For instance, BMP3 exhibits a restricted expression pattern *in vivo* [17], [19], [25], [39], [40], [41], [42], [43], [44], [45], [46], [47], [48], [49], [50], [51], [52], [53], [54] and its expression is modulated by several pathways [25], [41], [52], [55], [56], [57], [58], [59], [60], yet the *cis*-regulatory elements mediating these effects remain largely unknown. With this goal in mind, we sought to identify the *Bmp3* promoter in mouse *(M. musculus*) through functional and comparative genomic analyses. Having found that the minimal promoter resides within 0.8 kb upstream of *Bmp3* in *M. musculus*, we also identified a highly conserved element (ECR) upstream of the homologous *Bmp3* locus in every available eutherian mammal genome but not in any non-eutherian animal. We determined the minimal ECR that is present in all reference eutherian mammal genomes and identified the transcription factor binding sites conserved between *M. musculus*, rat (*R. norvegicus*), and human (*H. sapiens*). Collectively, our findings suggest that the high level of conservation of the *Bmp3* upstream ECR may preserve common *cis*-regulatory elements that govern *Bmp3* expression across eutherian mammals.

## Materials and Methods

### Plasmid construction

A series of plasmids containing fragments from the region upstream of *Bmp3* in *M. musculus* were generated from *M. musculus* genomic DNA using primer pairs as detailed in Table S1. For identification of the *Bmp3* minimal promoter, genomic fragments contained the first 63 nt of *Bmp3* exon 1 in order to include the annotated *Bmp3* transcription start site. For directional cloning into pGL4.14 (Promega), which is a promoter-less plasmid that encodes firefly luciferase, or pGL4.26 (Promega), in which firefly luciferase is under the control of the herpes simplex virus Thymidine Kinase minimal promoter, 5′ *Xho*I and 3′ *Hind*III restriction enzyme cut sites were appended to the genomic fragment by PCR. For directional cloning into pJL114, in which firefly luciferase is controlled upstream of the *Bmp3* minimal promoter in pJL114, 5′ *Sac*I and 3′ *Xho*I restriction enzyme cut sites were appended to the genomic fragment by PCR. Ligation was performed using DNA Ligation Kit (Takara) at 16°C for thirty minutes and transformed into OneShot TOP10 *E. coli* (Invitrogen) using the manufacturers′ protocol.

### Cell culture and *in vitro* experiments

HEK293T, UMR-106, and C2C12 cells were obtained from ATCC; primary calvarial osteoblasts were isolated from newborn wild type mice as described by Owen & Pan [61]. All cells were maintained in DMEM GlutaMAX (Gibco) supplemented with 10% FBS (Gibco).

For RT-PCR analyses, cells were scraped into PBS, centrifuged for 5 min at 500 x g at 4°C, the PBS aspirated, then cells were lysed and RNA collected using the RNeasy Mini Kit (QIAGEN) according to the manufacturer's protocol. cDNA was synthesized using the Transcriptor First Strand cDNA Synthesis Kit (Roche) according to the manufacturer's protocol. Newborn mouse hind limb RNA (collected as per animal protocol #04043 issued to VR with approval by the Harvard Medical Area Institutional Animal Care and Use Committee) served as a positive control. PCR on cDNA was performed using One*Taq* polymerase (NEB) according to the manufacturer's protocol. RT-PCR primers were designed to be complementary to *M. musculus*, *R. norvegicus*, and *H. sapiens* and cross exon boundaries (*Bmp3*: 5′-GGCTCTATGACAGGTACAGC-3′ and 5′-CTTTGGCATGGGGAACTGGCA-3′, *Hprt*: 5′-CCTGCTGGATTACATTAAAGCACTG-3′ and 5′-GTCAAGGGCATATCCAACAACAAAC-3′).

Luciferase activity was assayed using the Dual-Glo Luciferase Assay System (Promega). Cells were seeded at 3 k/cells per well in a 96-well plate. The next day, a plasmid encoding Renilla luciferase (pGL4.73, Promega) and test plasmids driving firefly luciferase were co-transfected into cells using XtremeGENE (Roche). After 48-hours, firefly and Renilla luciferase activity was quantified using a luminometer (LumiCount, Packard); expression of firefly luciferase under the control of the CMV promoter served as a positive control. Experiments were performed in triplicate or greater and are expressed as mean±SEM firefly luciferase/Renilla luciferase ratio. Data were normalized to the promoter-less (pGL4.14) or Thymidine Kinase minimal promoter (pGL4.26) control firefly luciferase plasmids.

### *In silico* experiments

DNA sequences were aligned using BLASTN [62] Version 2.2.26± or ECR Browser [63] through the respective online servers or locally using MUSCLE in MEGA5 software [64]. Accession number and region of DNA used for these analyses are denoted in the text and/or tables/figures. All analyses were performed between June and August 2012 using database versions current to that time period. The consensus *Bmp3* upstream minECR was constructed using the Los Alamos National Laboratory's Simple Consensus Maker (http://www.hiv.lanl.gov/content/sequence/CONSENSUS/consensus.html) using “Output aligned” parameter. For identification of transcription factor binding sites, DNA sequences were first aligned using zPicture [65] then transferred to rVista 2.0 [66]. Transcription factor affinity prediction was performed using TRAP [67], [68] using “transfac_2010.1 vertebrates” matrix, “mouse_promoters” background model, and Benjamini-Hochberg multiple test correction. DNA repeat motifs were identified using EMBL-EBI's CENSOR database [69] using the parameter “Mammalian.” Unless otherwise noted, all analyses were carried out using the default parameters.

### Statistical Analysis

Statistical significance was determined by One-way ANOVA with *post hoc* Newman-Keuls correction for multiple pairwise comparisons using GraphPad Prism. A *p* value of <0.05 was considered significant.

## Results

### Conservation analysis of the *Bmp3* upstream region

**Figure 1:**
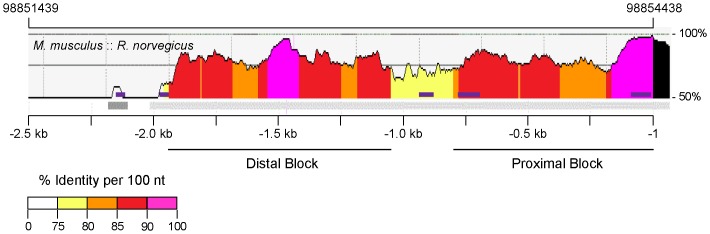
Conservation of the genomic region upstream of *Bmp3* in *M. musculus* and *R. norvegicus*. A: Composite image adapted from ECR Browser aligning the regions upstream of the *Bmp3* transcription start site between *M. musculus* and *R. norvegicus*. Threshold was set to ≥75% identity across sliding 100 nt window. This revealed a highly conserved 1.9 kb region proximal to the annotated transcription start site of *M. musculus Bmp3*. Within this region, there are two large blocks of ≥80% identity: a proximal one spanning from positions −1 to −806 and a distal one spanning positions −1057 to −1945. Each of these also contain a smaller region of ≥90% identity: −1 to −167 and −1408 and −1571, respectively. Genomic coordinates refer to location on *M. musculus* NC_000071.6. Gray bars indicate aligned segment. Purple boxes indicate repeat elements in *M. musculus*. Black region indicates 5′-end of *Bmp3* exon 1 in *M. musculus*.

To identify the *M. musculus Bmp3* promoter, we first used ECR Browser [63] to analyze the regions of high nucleotide conservation upstream of the *Bmp3* transcription start site between *M. musculus* and the closely related *R. norvegicus*. This revealed a high level of nucleotide identity (≥75% across sliding 100 nt window) in the approximately 1.9 kb region proximal to the annotated transcription start site of *M. musculus Bmp3* (Figure 1). Within this region, there are two large blocks of ≥80% identity: a proximal one spanning from positions −1 to −806 and a distal one spanning positions −1057 to −1945. Each of these also contains a smaller region of ≥90% identity: −1 to −167 and −1408 and −1571, respectively. The nucleotide identity between *M. musculus* and *R. norvegicus* drops sharply upstream of this region, becoming more conserved once again beyond 3.2 kb upstream; this poorly conserved region is also present when aligning *M. musculus* and the alternate *R. norvegicus* reference genome, indicating this finding is not due to an assembly error (JWL, data not shown).

### Functional identification of the *M. musculus Bmp3* promoter and an upstream repressive element

**Figure 2:**
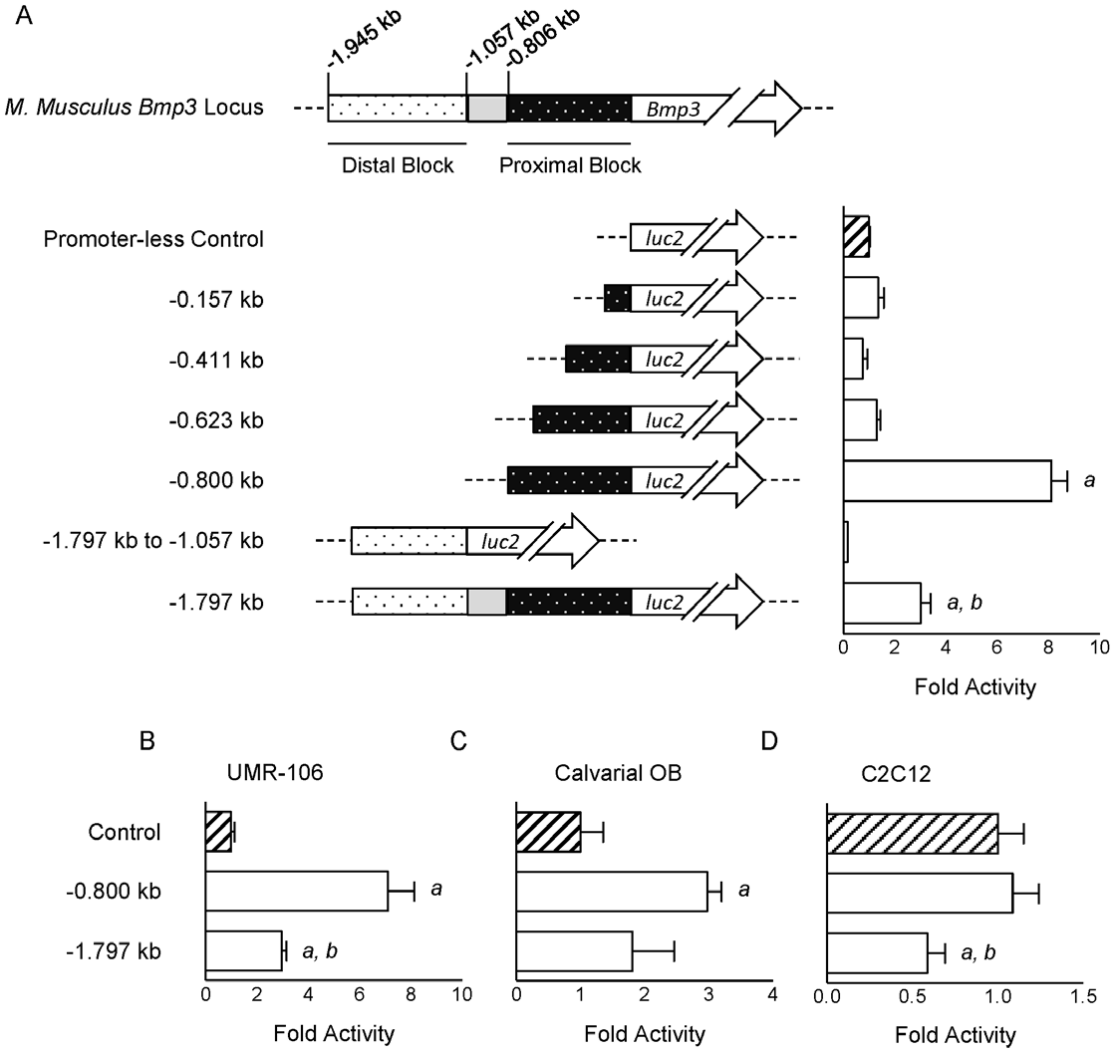
Functional characterization of the genomic region upstream of *M. musculus Bmp3*. A–D: Regulation of firefly luciferase activity driven by fragments from the region upstream of *M. musculus Bmp3* in HEK293T cells (**A**), UMR-106 cells (**B**), primary calvarial osteoblasts (**C**), and C2C12 cells (**D**). All data are mean±SEM normalized to promoter-less control. *p*<0.05 as determined by One-way ANOVA with *post hoc* Newman-Keuls correction is indicated by “*a*” versus promoter-less control and by “b” versus −0.800 kb fragment.

Due to the close evolutionary-relatedness of *M. musculus* and *R. norvegicus*, we predicted that the *M. musculus Bmp3* promoter would reside within the conserved region that abuts the *Bmp3* transcription start site instead of farther upstream in the poorly conserved region. We established a reporter system using HEK293T cells wherein firefly luciferase expression is controlled by fragments from the putative *M. musculus Bmp3* promoter. We first confirmed that HEK293T cells express *Bmp3* basally (Figure S1A), making them a suitable system in which to study the *Bmp3* promoter. Driving firefly luciferase expression by increasingly larger fragments of the putative *Bmp3* promoter demonstrated that the proximal, highly conserved block 0.800 kb upstream of *Bmp3* is the minimal region necessary for expression (Figure 2A). To evaluate the potential action of this genomic region in osteoblasts, we utilized the osteoblast-like UMR-106 osteosarcoma cell line [70] and primary mouse calvarial osteoblasts, both of which express *Bmp3* basally (Figure S1B-C and [60]). Consistent with our findings in HEK293T cells, the 0.800 kb upstream of *Bmp3* is sufficient to drive firefly luciferase expression in these cells (Figure 2B–C). Specific promoter activity of the 0.800 kb region was demonstrated by its inability to drive firefly luciferase expression in C2C12 myoblast cells (Figure 2D), which do not express *Bmp3* (Figure S1D). rVista2.0 [66] analysis of this 800 nt region (hereafter referred to as the *Bmp3* minimal promoter) identified binding sites for a number of general (eg, TFII-I) and pathway-specific transcription factors (eg, SMAD, TCF/LEF, AP-1, STAT, and KF-kappaB), many of which are conserved with *R. norvegicus* (Table S2). We then performed TRanscription factor Affinity Prediction (TRAP) analyses [67], [68] to examine each predicted site based upon strength of binding affinity (Table S2).

Having identified the minimal promoter for *M. musculus Bmp3*, we turned our attention to the distal conserved block and intervening sequence (IvS) between the distal and proximal blocks (Figure 1 and Figure 2A). We did not observe promoter activity when attempting to drive firefly luciferase expression with the highest conserved portion of the distal block (Figure 2A). Rather, appending the distal block and IvS to the *Bmp3* minimal promoter reduced the promoter activity (Figure 2A), indicating that a portion of this ∼1 kb region upstream of the minimal promoter acts as a basal repressive element in HEK293T cells. In support of this finding, rVista 2.0 analysis identifies binding sites for a number of potential repressive transcription factors (Table S2). To determine if the repressive action of this region is specific to HEK293T cells, we examined its function in UMR-106 cells, primary mouse calvarial osteoblasts, and C2C12 cells. In each cell type, the ∼1 kb region upstream of the *Bmp3* minimal promoter acted as a repressive element, though this did not reach statistical significance in primary osteoblasts (Figure 2B–D).

### Identification of an evolutionary conserved region (ECR) upstream of *Bmp3* in mammals

The high degree of identity in the distal block/IvS between *M. musculus* and *R. norvegicus* raises the possibility that this could be an evolutionary conserved region (ECR) that regulates the expression of *Bmp3*. However, the overall level of conservation between *M. musculus* and *R. norvegicus* is too high to allow us to make this conclusion. For this reason, we extended our nucleotide conservation analysis by performing pairwise alignments of the *Bmp3* upstream regions between *M. musculus* and more distantly related species. We were unable to use ECR Browser for these analyses as the species that are aligned to *M. musculus* in this database are limited. Instead, we performed alignments using BLASTN in sequential 500 nt sections from *M. musculus* against the full-length 5 kb region upstream of *Bmp3* from the other species. This approach also allowed for the possibility of genomic insertions or deletions. To ensure the fidelity and accuracy of these analyses, we focused our attention on the thirty-nine NCBI Reference Sequence (RefSeq) animal genomes, at least twenty-eight of which contain an annotated *Bmp3* or *Bmp3-like* gene.

**Figure 3:**
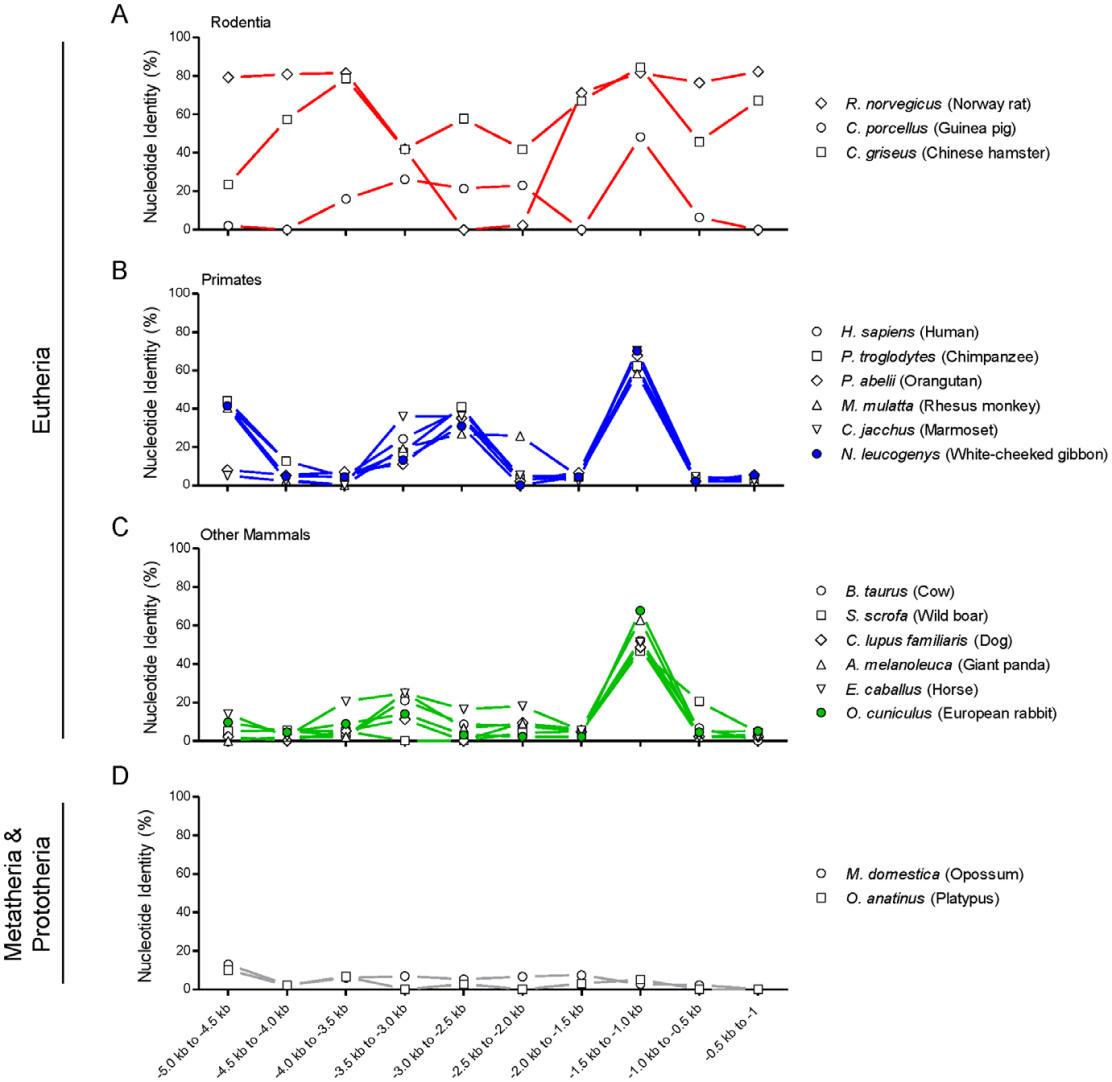
Conservation of the region upstream of *Bmp3* between *M. musculus* and RefSeq mammal genomes. Pairwise alignments were performed between the *Bmp3* upstream regions of *M. musculus* and all seventeen available RefSeq mammalian genomes (15 eutherian, 2 non-eutherian) using BLAST 2.2.26+. Comparing sequential 500 nt sections from *M. musculus* against the full-length 5 kb region upstream of *Bmp3* from the other species allowed for the possibility of genomic insertions or deletions. Findings are separated into taxonomic classification for clarity (**A**–**D**). Actual numbers for these analyses are listed in Table S3. Conservation with *R. norvegicus*, *H. sapiens*, and *B. taurus* are based upon the primary assembly.

As proof of principle, the highest identity to *M. musculus* was found among the other two muroideans, *R. norvegicus* and Chinese hamster (*C. griseus*) (Figure 3A and Table S3). Strikingly, among mammals more distantly related to *M. musculus*, we found the highest degree of conservation when using a portion of the distal conserved block upstream of *Bmp3* in *M. musculus* (Figure 3A–C and Table S3). This pattern was present in every eutherian mammal in our cohort (15/15), but in neither of the non-eutherian mammals, *M. domestica* and *O. anatinus* (Figure 3D and Table S3), nor in any of the twenty-two non-mammalian RefSeq genomes (JWL, data not shown).

**Figure 4:**
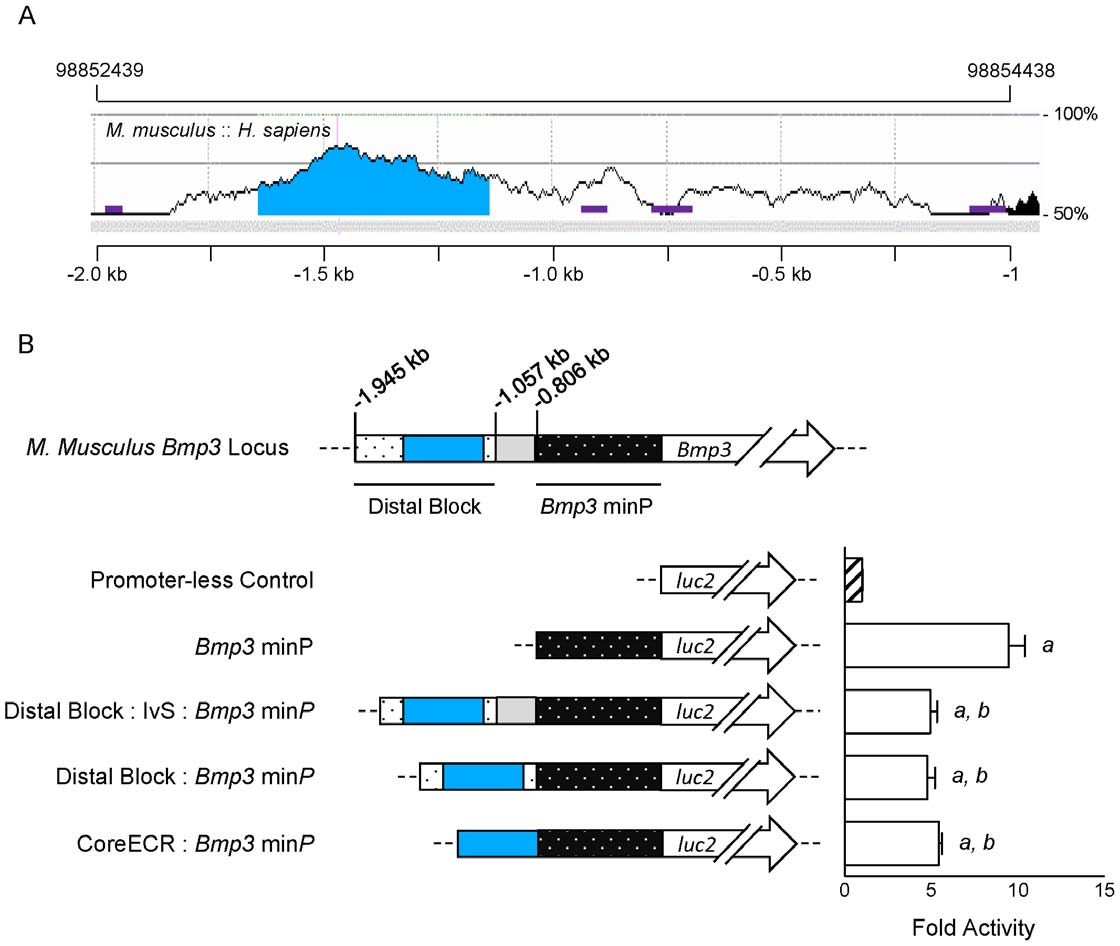
Identification of a repressive CoreECR upstream of *Bmp3 between M. musculus* and *H. sapiens*. A: Image adapted from ECR Browser aligning the regions upstream of the *Bmp3* transcription start site between *M. musculus* and *H. sapiens*. Threshold was set to ≥77% identity across sliding 350 nt window. This revealed a CoreECR upstream of the annotated transcription start site of *Bmp3* in both species (blue region –*M. musculus*: 98852797–98853301 on NC_000071.6; *H. sapiens*: 81949936–81950336 on NC_000004.11). Genomic coordinates refer to location on *M. musculus* NC_000071.6. Gray bar indicates aligned segment. Purple boxes indicate repeat elements in *M. musculus*. Black region indicates 5′-end of *Bmp3* exon 1 in *M. musculus*. B: Regulation of firefly luciferase activity driven by fragments from the region upstream of *M. musculus Bmp3* in HEK293T cells. minP = minimal promoter; IvS = intervening sequence between distal and proximal conserved blocks. Data are mean±SEM normalized to promoter-less control. *p*<0.05 as determined by One-way ANOVA with *post hoc* Newman-Keuls correction is indicated by “*a*” versus promoter-less control and by “b” versus −0.800 kb fragment.

These findings suggested that all or a portion of the distal block conserved between *M. musculus* and *R. norvegicus* upstream of *Bmp3* is an ECR among eutherian mammals. To test this directly, we first aligned the region upstream of *Bmp3* in *M. musculus* and *H. sapiens* using ECR Browser [63]. Setting our threshold at 77% identity across a sliding 350 nt window to pinpoint lengthy, highly conserved “CoreECRs” [63], we identified a 505 nt region in *M. musculus* spanning from position −1642 to −1138 upstream of the *Bmp3* transcription start site that is within the distal block conserved between *M. musculus* and *R. norvegicus* (Figure 4A). The absolute position of the CoreECR is 98852797–98853301 on NC_000071.6. This is the only CoreECR conserved between *M. musculus* and *H. sapiens* within 7.6 kb upstream and 30 kb downstream of the *Bmp3* locus (JWL, data not shown).

To determine the function of the *Bmp3* CoreECR, we generated plasmids in which portions of the repressive distal block/IvS region were placed upstream of the *Bmp3* minimal promoter. This revealed that the CoreECR is as effective as the complete ∼1 kb region in its ability to repress the *Bmp3* minimal promoter (Figure 4B).

We then used BLASTN to align the *M. musculus*: *H. sapiens* CoreECR sequence to all thirty-nine available animal RefSeq genomes, revealing significant conservation of this sequence in all (15/15) eutherian mammals (Table S4); in each, the conserved region was upstream of *Bmp3*. Moreover, although the *Bmp3* gene has been found in at least eleven of the non-eutherian animals in the RefSeq genome database, the *Bmp3* upstream ECR was not found in any of the twenty-four non-eutherian animal RefSeq genomes analyzed (JWL, data not shown).

### Determination of the minimal *Bmp3* upstream ECR (minECR)

**Figure 5:**
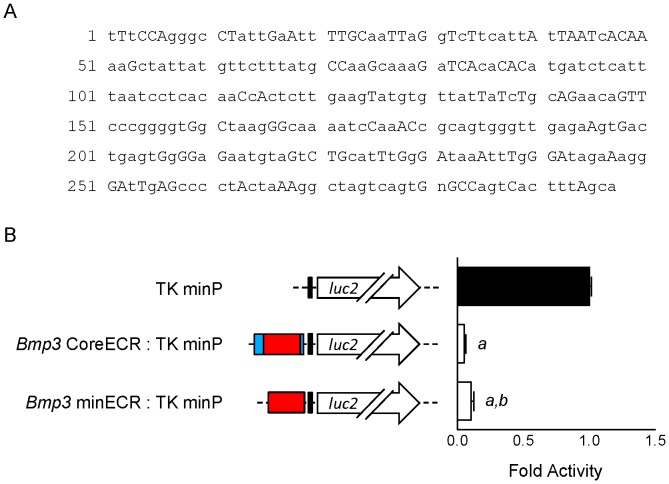
Examination of the minimal *Bmp3* upstream ECR (minECR) present in all RefSeq mammals. BLASTN (Version 2.2.26+) was used to align the *M. musculus*: *H. sapiens* CoreECR sequence to all sixteen available eutherian mammal RefSeq genomes in order to identify the *Bmp3* upstream ECR shared with *M. musculus*. Each full-length ECR was then aligned using MUSCLE in MEGA5 [64], identifying a minimal *Bmp3* upstream ECR (minECR) with a consensus length of 297 nt. The mean identity to the consensus minECR is 90% (range: 79%–97%, median: 92%); the individual nucleotide conservation is shown in Figure S2. Uppercase letters in the consensus sequence indicate 100% conservation, while lowercase letters indicate the majority nucleotide; “n” indicates no consensus nucleotide could be determined. B: Regulation of Thymidine Kinase minimal promoter (TK minP) by fragments from the region upstream of *M. musculus Bmp3* in HEK293T cells. Data are mean±SEM normalized to promoter-less control. *p*<0.05 as determined by One-way ANOVA with *post hoc* Newman-Keuls correction is indicated by “*a*” versus promoter-less control and by “b” versus CoreECR fragment.

We have demonstrated that an ECR shared with *M. musculus* lies upstream of *Bmp3* in each eutherian mammal in the RefSeq genome database. However, the total length and identity of the *Bmp3* upstream ECR varies (Table S4), prompting us to determine the minimal ECR that is conserved across all eutherian mammals in the RefSeq cohort. To do so, the full-length ECRs from each species were aligned using MUSCLE in MEGA5 [64], identifying a minimal *Bmp3* upstream ECR (minECR) with a consensus length of 297 nt (Figure 5). The mean identity to the consensus minECR is 90% (range: 79%–97%, median: 92%) (Table 1); the individual nucleotide conservation is shown in Figure S2.

**Table 1:** Conservation of the consensus minimal *Bmp3* upstream Evolutionary Conserved Region (minECR) in RefSeq animal genomes.

		Accession Number	minECR		Distance from *Bmp3* (nt)	
			Location	% Identity	5′	3′
**Rodentia**	*M. musculus*	NC_000071.6	95552942.95553250	79%	−1497	−1189
	*R. norvegicus*	NC_005113.3	12332541.12332253, complement	82%	−1511	−1223
		AC_000082.1	10827337.10827049, complement	82%	−1511	−1223
	*C. porcellus*	NT_176414.1	5088909.5089198	80%	−1789	−1500
	*C. griseus*	NW_003616697.1	55307.54999, complement	82%	−1611	−1303
**Primates**	*H. sapiens*	NC_000004.11	81950009.81950305	97%	−2110	−1814
		AC_000136.1	77693899.77694195	97%	−2111	−1815
	*P. troglodytes*	NC_006471.3	49002602.49002306, complement	97%	−2101	−1805
	*P. abelii*	NC_012595.1	84489849.84490145	97%	−2120	−1824
	*M. mulatta*	NC_007862.1	48535985.48535688, complement	96%	−2137	−1840
	*N. leucogenys*	NW_003501411.1	13030680.13030976	96%	−2109	−1813
	*C. jacchus*	NC_013898.1	113372870.113372574, complement	96%	−2772	−2476
**Other Orders**	*B. taurus*	AC_000163.1	97583173.97583469	93%	−2360	−2064
		NC_007304.5	99175053.99175349	93%	−2360	−2064
	*S. scrofa*	NC_010450.3	146200934.146200644, complement	78%	−1914	−1624
	*C. lupus familiaris*	NC_006614.2	8169902.8170198	87%	−2135	−1839
	*A. melanoleuca*	NW_003217292.1	2528248.2527953, complement	92%	−2167	−1872
	*E. caballus*	NC_009146.2	55766669.55766369, complement	90%	−741	−441
	*O. cuniculus*	NC_013683.1	69240613.69240317, complement	86%	−2347	−2051

Species are separated by taxonomic order. Distance from *Bmp3* is calculated from the annotated transcription start site. nt: nucleotide.

The relative genomic location of the *Bmp3* upstream minECR varies from species to species, but is quite consistent among closely-related species (Table 1). We found the 5′-end of the *Bmp3* upstream minECR to be as close as position −741 in *E. caballus* and as distant as position −2772 in *C. jacchus* (Table 1). To determine if the minECR retains the repressive activity of the full-length CoreECR, we placed both sequences upstream of the Thymidine Kinase minimal promoter. This revealed that the both were capable of repressing the Thymidine Kinase minimal promoter, but the activity of the longer CoreECR was slightly stronger than the minECR (Figure 5B).

We analyzed the consensus minECR sequence using CENSOR [69] to identify potential DNA repeat elements, which revealed a reverse-orientation, partial match to the Short Interspersed Element (SINE) MIRb (Figure S2). However, inspection of the minECR from each individual species shows that this partial repeat is predominantly found in primates, and it resides in one of the more poorly aligned regions of the consensus minECR- the mean identity to this 57 nt region is 88%, and removing it from the consensus minECR increases the overall identity in 11/16 species, raising the mean identity to 91%. For this reason, and the small size of this partial repeat region relative to the full minECR, we do not credit the high conservation of the *Bmp3* upstream minECR across eutherian mammals to a conserved retrotransposon.

Additionally, we confirmed that the *Bmp3* minECR resides in a non-coding region of the genome by performing BLASTN alignment of the consensus *Bmp3* upstream minECR against the database of GenBank/EMBL/DDBJ expressed sequence tags (ESTs), which comprise >73,580,051 sequences. This failed to identify any EST with significant alignment to the consensus *Bmp3* upstream minECR- the highest identity was 85% over 59 nt (JWL, data not shown). For comparison, exon 1 of *M. musculus Bmp3* readily aligned to multiple ESTs from *M. musculus* and other species (JWL, data not shown).

### Identification of the consensus *Bmp3* upstream minECR in all available eutherian mammal genomes

Our findings suggest that the *Bmp3* upstream ECR is a *cis*-regulatory element unique to eutherian mammals. However, since the number of RefSeq genomes is fairly small at present, we extended our investigation to species for which a RefSeq genome is not available. We used BLASTN to align the consensus *Bmp3* upstream minECR to the whole-genome shotgun sequence database, which comprises 191 animal species. This revealed significant conservation of the *Bmp3* upstream minECR in thirty-one of thirty-nine eutherian mammals that were not represented by the RefSeq cohort (Table S5); notably, at the present stage of their assembly, there is no identifiable *Bmp3* gene in any of the eight eutherian mammals in which the *Bmp3* upstream ECR was not identified. Moreover, although the *Bmp3* gene has been found in at least sixteen non-eutherian animals in this databse, the *Bmp3* upstream minECR was not found in any of the 136 non-eutherian animal genomes analyzed (JWL, data not shown).

### Identification of transcription factor binding sites in the consensus *Bmp3* upstream ECR

Next, we turned our focus to examining the potential *cis*-regulatory role(s) played by the *Bmp3* upstream ECR. To do so, we first used rVista 2.0 to identify the transcription factor binding sites (TFBSs) in the consensus *Bmp3* minECR sequence (Table S6). This yielded a list of fifty-one distinct binding sites, the majority of which are estimated to be high affinity by TRAP analysis and are pathway-specific transcription factors (eg, C/EBP, Ikaros, AP1).

**Figure 6:**
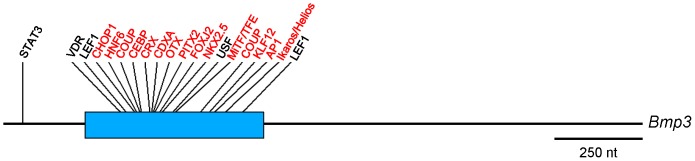
Transcription factor binding sites conserved between *M. musculus* and *H. sapiens in the Bmp3* upstream region. Transcription factor binding sites in the 1.797 kb genomic fragment were identified using rVista 2.0 for *M. musculus* and *H. sapiens*. With the exception of one, all transcription factor binding sites within 2 kb upstream of *Bmp3* that are conserved between *M. musculus* and *H. sapiens* lie within the CoreECR. Transcription factor binding sites in red are also conserved with the consensus *Bmp3* upstream minECR. Drawn to scale using the *M. musculus* 1.797 kb genomic fragment from Figure 1.

Finally, we sought to validate the evolutionary conservation of the *Bmp3* upstream minECR as a cluster of *cis*-regulatory elements by determining the degree to which conserved *cis*-regulatory elements exist outside of the minECR. To do so, we aligned the regions upstream of *M. musculus* and *H. sapiens*, then identified conserved transcription factor binding sites using rVista 2.0 (Table S7). This revealed that, with the exception of one (STAT3), all of the transcription factor binding sites within 2 kb upstream of *Bmp3* in *M. musculus* that are conserved with *H. sapiens* reside within the *M. musculus*: *H. sapiens* CoreECR (Figure 6 and Table S7). Moreover, sixteen of the twenty (80%) transcription factor binding sites conserved between *M. musculus* and *H. sapiens* upstream of *Bmp3* reside within the limits of the minECR, and all but one (USF) are present in the consensus minECR sequence (Figure 6 and Table S7). For secondary confirmation of this finding, we aligned the regions upstream of *Bmp3* from *M. musculus, R. norvegicus, C. griseus*, and Guinea pig (*C. porcellus*). This revealed that all of the TFBSs within 3.8 kb upstream of *Bmp3* that are conserved across the RefSeq rodent genomes (9/9) reside within the limits of the minECR and are present in the consensus minECR sequence (JWL, data not shown). These findings indicate that the consensus minECR could serve as a valid predictor of highly conserved *cis*-regulatory elements that govern *Bmp3* expression.

## Discussion

In the present study, we took a comparative genomics approach to identify potential *cis*-regulatory elements controling *Bmp3* expression in *M. musculus*. Functional characterization of various genomic fragments revealed the 0.8 kb region proximal to the annotated *M. musculus Bmp3* transcription start site to be the minimal promoter in HEK293T cells, UMR-106 osteosarcoma cells, and primary calvarial osteoblasts. This region corresponds to a highly conserved block (≥80% identity across a sliding 100 nt window) that is shared between *M. musculus* and *R. norvegicus* and contains binding sites for a number of both general and pathway-specific transcription factors. We analyzed the 5 kb upstream region and exon 1 of *Bmp3* from *M. musculus* using Neural Network Promoter Scan [71] to identify potential transcription starts sites (TSSs) within the minimal promoter. This revealed two TSSs (−0.108 kb and −0.452 kb) upstream of exon 1. These two TSSs, in addition to the annotated TSS, are contained in our luciferase reporter plasmids. Thus, we are unable to determine which TSS is used in each cell type; however, the 0.8 kb fragment consistently led to induction of luciferase expression in each of our assays whereas shorter fragments did not, leading us to conclude that we have identified the minimal region necessary to drive *Bmp3* expression. Our findings are consistent with a previous report that attained promoter activity using the 2 kb region upstream of *Bmp3* in *R. norvegicus* [25].

We were surprised to find that the *Bmp3* minimal promoter is poorly conserved between *M. musculus* and *H. sapiens*, although this finding is consistent with a previous report comparing the promoter for *Bmp3b* (also known as GDF10) between *M. musculus* and *H. sapiens* [9]. While *Bmp3b* likely arose from duplication of the *Bmp3* gene, or vice versa [9], [72], alignment of the 5 kb regions upstream of *Bmp3* and *Bmp3b* in *M. musculus* failed to demonstrate any significant nucleotide identity (JWL, data not shown), suggesting that *Bmp3* and *Bmp3b* have evolved unique mechanisms regulating their expression. This idea is supported by the fact that the spatio-temporal expression domains of *Bmp3* and *Bmp3b* differ quite drastically [72].

After identifying the proximal block conserved between *M. musculus* and *R. norvegicus* as the minimal promoter, we turned our attention to the remaining highly conserved region (−1.9 kb to −0.8 kb upstream of *Bmp3*). Interestingly, appending this region to the minimal promoter repressed promoter activity. Fidelity of the genomic fragment in this reporter plasmid was confirmed by bi-directional sequencing and our finding was consistent in each repetition of our assay, leading us to conclude that this region is capable of repressing basal *Bmp3* expression in each cell type tested. This is supported by the fact that, when we examined potential TFBSs in this region by rVista2.0 [66] analysis, we identified binding sites for a number of potential repressive transcription factors.

BLASTN alignment of the 5 kb regions between *M. musculus* and each of the other thirty-nine complete RefSeq genomes revealed that a portion of the distal block was conserved with every eutherian mammal (15/15), but not in any non-eutherian species (0/24) even though a *Bmp3* or *Bmp3-like* locus has been annotated for at least thirteen non-eutherian species (eg, *X. tropicalis, D. rerio, G. gallus, O. anatinus, M. domestica*). We went on to narrow this conserved region to 297 nt that is shared between all eutherian mammals in the RefSeq cohort, and then found this minimal ECR in thirty-one additional eutherian mammals represented in the whole-genome shotgun sequence (WGS) database (total of forty-seven eutherian mammals between RefSeq and WGS databases). As with the RefSeq database, we did not find the minimal ECR in any of the 136 non-eutherian animal genomes in the WGS database. Of note, genomic sequences are presently available from only four non-eutherian mammals (*O. anatinus, M. domestica, M. eugenii* and *S. harissii*). Thus, we conclude from our findings that the *Bmp3* upstream ECR is a eutherian mammal-specific *cis*-element, but are aware that future studies are required to definitively show if this ECR is also found in metatherian or prototherian mammals.

The *Bmp3* upstream minECR is a highly conserved genomic region near the minimal promoter that represses basal promoter activity. This arrangement is similar to the conserved, high GC-content short-range repressive elements that have been described near the *Bmp2* promoter [73] –though the *Bmp3* upstream minECR bears no alignment to these regions and the GC content is only 45% (JWL, data not shown). To examine possible regulatory mechanisms, we examined the *Bmp3* upstream minECR using rVista 2.0, which not only pinpoints consensus TFBSs using the TRANSFAC database but combines this information with sequence conservation analyses of the surrounding 20 nt to identify the most biologically relevant TFBSs [66], and TRAP analysis, which predicts transcription factor binding affinity to each site [67], [68]. This revealed that sixteen of the twenty (80%) transcription factor binding sites conserved between *M. musculus* and *H. sapiens* upstream of *Bmp3* reside within the limits of the minECR, and the majority of these are predicted to be high-affinity binding sites. Moreover, even among the more closely-related rodents *M. musculus, R. norvegicus, C. griseus*, and *C. porcellus* we found that all of the TFBSs within 3.8 kb upstream of *Bmp3* (9/9) reside within the limits of the minECR (JWL, data not shown).

Our identification of a highly conserved block of potential *cis*-regulatory elements upstream of *Bmp3* in mammals provides a foundation for future studies examining modulation of *Bmp3* expression. In support of this, the TFBSs that we identified are highly consistent with what has previously been reported on the regulation of *Bmp3*. For instance, binding sites for HNF1, VDR, AP1, and NF-kappaB, all of which have been shown to regulate *Bmp3* expression [25], [52], [55], [57], [60], are present in the *Bmp3* upstream minECR.

Of particular interest to us is the role of *Bmp3* in regulation of bone formation. Similar to many osteogenic BMP ligands, *Bmp3* is expressed in osteoblasts [17], [45], [46], [53], [54]. However, while canonical BMP signaling is required for bone formation (reviewed in [74]), *Bmp3* knockout mice have high bone mass [15] and overexpression of BMP3 leads to spontaneous rib fractures in mice [22], indicating that BMP3 is a negative regulator of osteogenesis. As such, the identification of a highly-conserved repressive element near the *Bmp3* promoter could determine mechanisms to reduce *Bmp3* expression in diseases of low bone mass such as osteopenia and osteoporosis.

## Concluding Remarks

We identified the minimal *Bmp3* promoter from *M. musculus* and determined that this region is highly conserved with *R. norvegicus*. We also found that a highly conserved upstream region abutting the minimal promoter is able to repress the minimal promoter. A portion of this region is conserved among all available eutherian mammal genomes (47/47), but not in any non-eutherian animal (0/136). We also identified multiple conserved transcription factor binding sites in the *Bmp3* upstream ECR. Collectively, these findings suggest that the high level of conservation of the *Bmp3* upstream ECR may preserve common *cis*-regulatory elements that govern *Bmp3* expression across eutherian mammals.

## Supporting Information

Figure S1 ***Bmp3* expression analysis.** RT-PCR for *Bmp3* in HEK293T cells (**A**), UMR-106 cells (**B**), primary mouse calvarial osteoblasts(cOBs, C), and C2C12 cells 9D) compared to *Hprt* housekeeping control. Newborn mouse hind limb cDNA was used as a positive control in all experiments (only shown in **A**). Intervening lanes from a single gel removed in A (indicated by white bar).
(TIFF)

Figure S2 **Determination and individual nucleotide conservation of the minimal *Bmp3* upstream ECR (minECR) present in all RefSeq mammals.** BLASTN (Version 2.2.26+) was used to align the *M. musculus*: *H. sapiens* CoreECR sequence to all sixteen available eutherian mammal RefSeq genomes in order to identify the *Bmp3* upstream ECR shared with *M. musculus*. Each full-length ECR was then aligned using MUSCLE in MEGA5 [64], identifying a minimal *Bmp3* upstream ECR (minECR) with a consensus length of 297 nt (319 nt as shown when including insertions found in some species). The consensus sequence was determined using Los Alamos National Laboratory's Simple Consensus Maker. Uppercase letters in the consensus sequence indicate 100% conservation, while lowercase letters indicate the majority nucleotide. “n” indicates no consensus nucleotide could be determined and “.” indicates a gap. For each individual species, a dash indicates a match to the consensus, while “A, T, C, or G” indicates a mismatch to the consensus. The mean identity to the consensus minECR is 90% (range: 79%–97%, median: 92%). A partial match to the SINE2-type repeat MIRb, found primarily in primates, is denoted in red.
(TIFF)

Table S1 Primers used for firefly luciferase reporter plasmid construction.
(XLSX)

Table S2 Transcription factor binding sites upstream of *M. musculus Bmp3*.
(XLSX)

Table S3 Nucleotide alignments of the *Bmp3* 5 kb upstream regions between *M. musculus* and other mammals.
(XLS)

Table S4 Conservation analysis of the *Bmp3* upstream Evolutionary Conserved Region (ECR) in RefSeq animal genomes.
(XLS)

Table S5 Conservation analysis of the minimal *Bmp3* upstream Evolutionary Conserved Region (minECR) in all available animal genomes.
(XLS)

Table S6 Transcription factor binding sites in the consensus minimal *Bmp3* upstream ECR and conservation in Rodents/Primates.
(XLSX)

Table S7 Transcription factor binding sites conserved between *M. musculus* and *H. sapiens* in the region upstream of *Bmp3*.
(XLS)
