# Supplementary figures and images for: Comparative Genomics Identifies the Mouse Bmp3 Promoter and an Upstream Evolutionary Conserved Region (ECR) in Mammals
Source: PLoS One. 2013 Feb 22;8(2):e57840. doi: 10.1371/journal.pone.0057840 (PMC3579780; doi:10.1371/journal.pone.0057840)

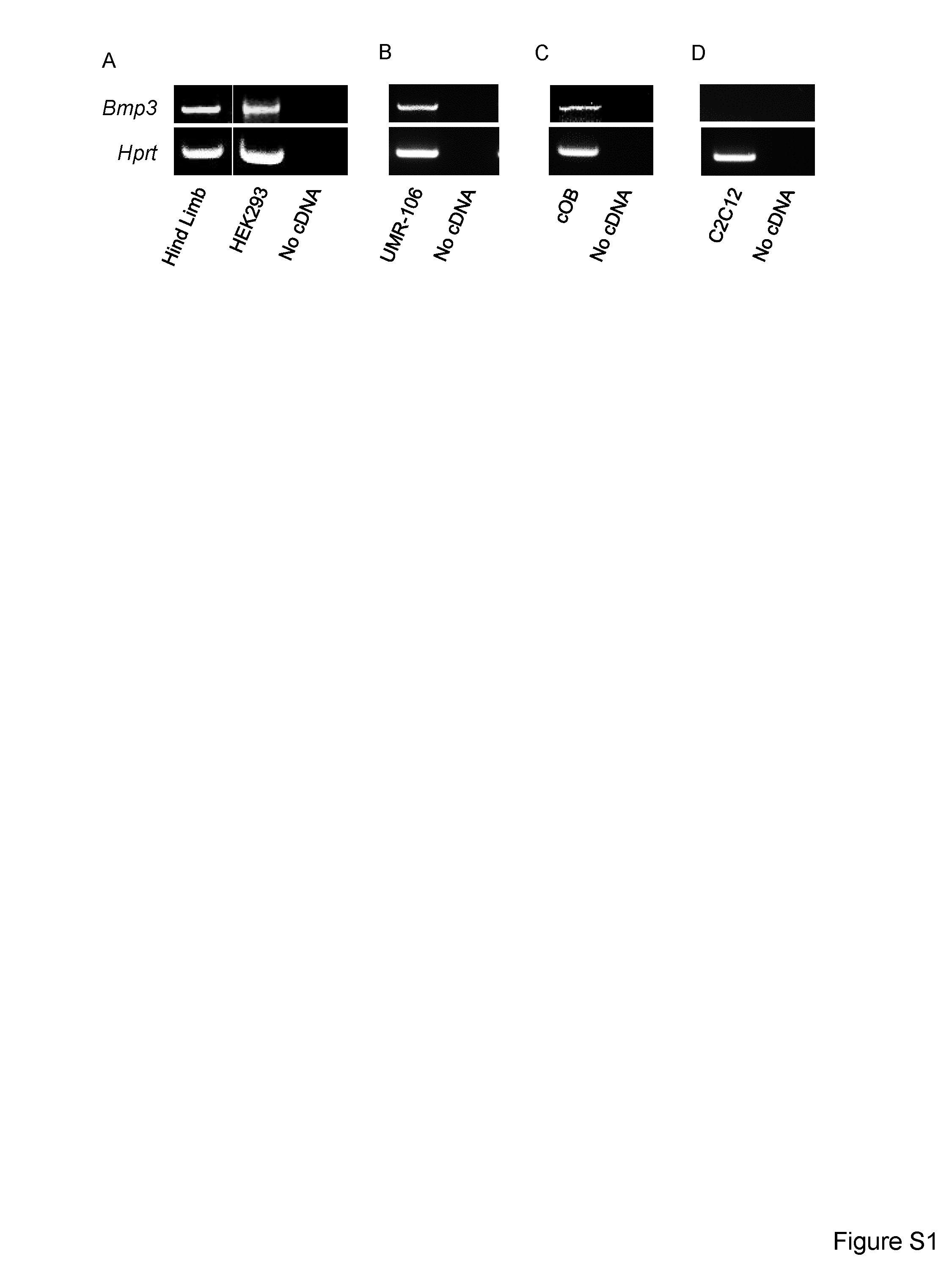

Supplement: Figure S1 — Bmp3 expression analysis. RT-PCR for Bmp3 in HEK293T cells (A), UMR-106 cells (B), primary mouse calvarial osteoblasts(cOBs, C), and C2C12 cells 9D) compared to Hprt housekeeping control. Newborn mouse hind limb cDNA was used as a positive control in all experiments (only shown in A). Intervening lanes from a single gel removed in A (indicated by white bar). (TIFF) [file pone.0057840.s001.tif]

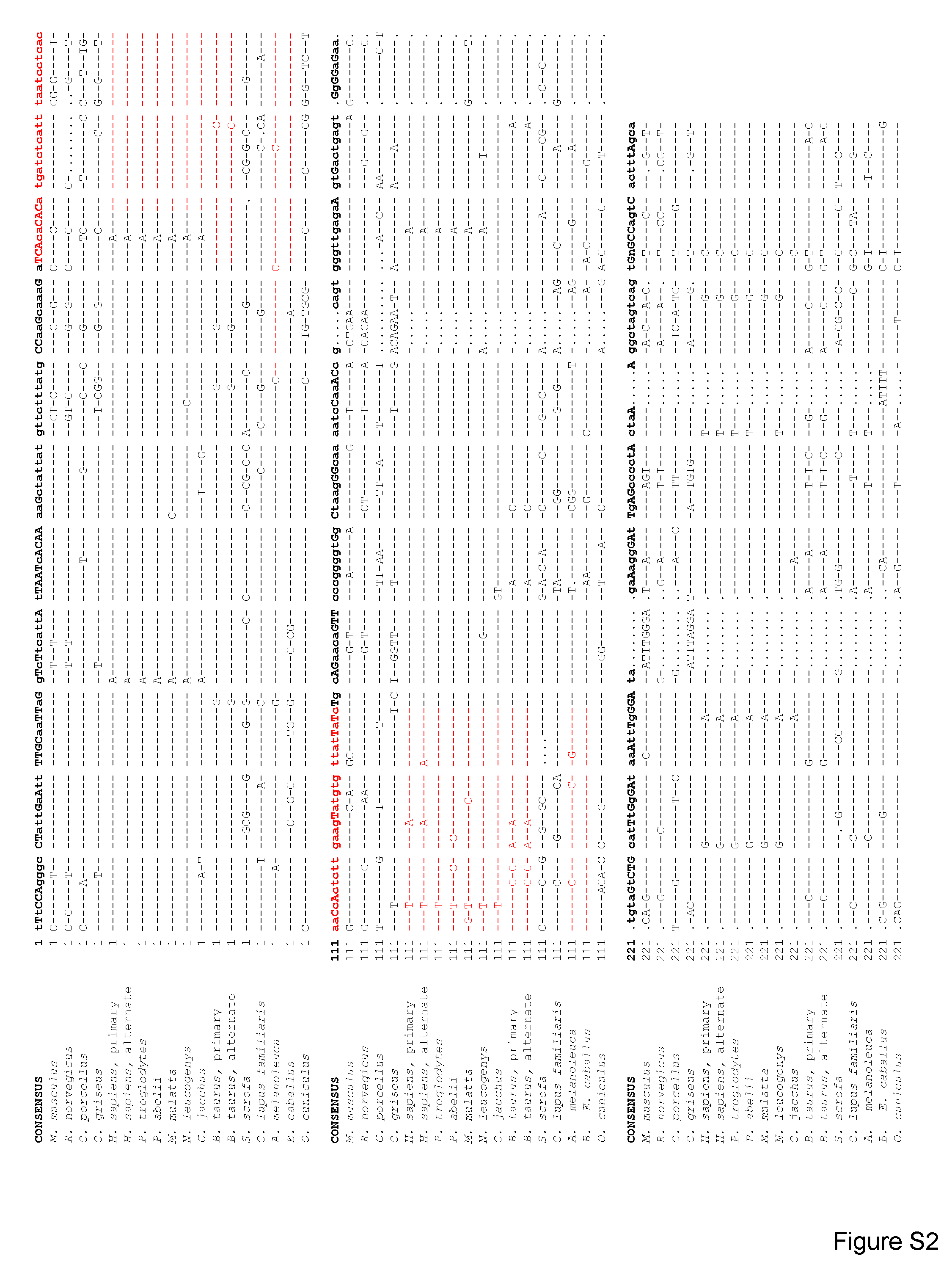

Supplement: Figure S2 — Determination and individual nucleotide conservation of the minimal Bmp3 upstream ECR (minECR) present in all RefSeq mammals. BLASTN (Version 2.2.26+) was used to align the M. musculus: H. sapiens CoreECR sequence to all sixteen available eutherian mammal RefSeq genomes in order to identify the Bmp3 upstream ECR shared with M. musculus. Each full-length ECR was then aligned using MUSCLE in MEGA5 [64], identifying a minimal Bmp3 upstream ECR (minECR) with a consensus length of 297 nt (319 nt as shown when including insertions found in some species). The consensus sequence was determined using Los Alamos National Laboratory's Simple Consensus Maker. Uppercase letters in the consensus sequence indicate 100% conservation, while lowercase letters indicate the majority nucleotide. “n” indicates no consensus nucleotide could be determined and “.” indicates a gap. For each individual species, a dash indicates a match to the consensus, while “A, T, C, or G” indicates a mismatch to the consensus. The mean identity to the consensus minECR is 90% (range: 79%–97%, median: 92%). A partial match to the SINE2-type repeat MIRb, found primarily in primates, is denoted in red. (TIFF) [file pone.0057840.s002.tif]
